# The evolving role of reduced-dose total skin electron beam therapy in skin malignancies: the renaissance of a rare indication

**DOI:** 10.1007/s00066-023-02115-4

**Published:** 2023-07-14

**Authors:** Khaled Elsayad, Hans Theodor Eich

**Affiliations:** Department of Radiation Oncology, University Hospital of Muenster, Building A1, 1 Albert Schweitzer Campus, 48149 Münster, Germany

**Keywords:** Radiotherapy, Kaposi sarcoma, Cutaneous lymphoma, Metastasis, Leukemia cutis

## Abstract

Definitive radiation therapy is an effective local treatment for several cutaneous malignancies. Patients with diffuse or generalized skin manifestations might require total skin electron beam therapy (TSEBT) as an alternative treatment to the chasing technique. In this short communication, we highlight the evolving role of TSEBT and present its role in various forms of skin malignancies.

## Radiotherapy technique

**Fig. 1:**
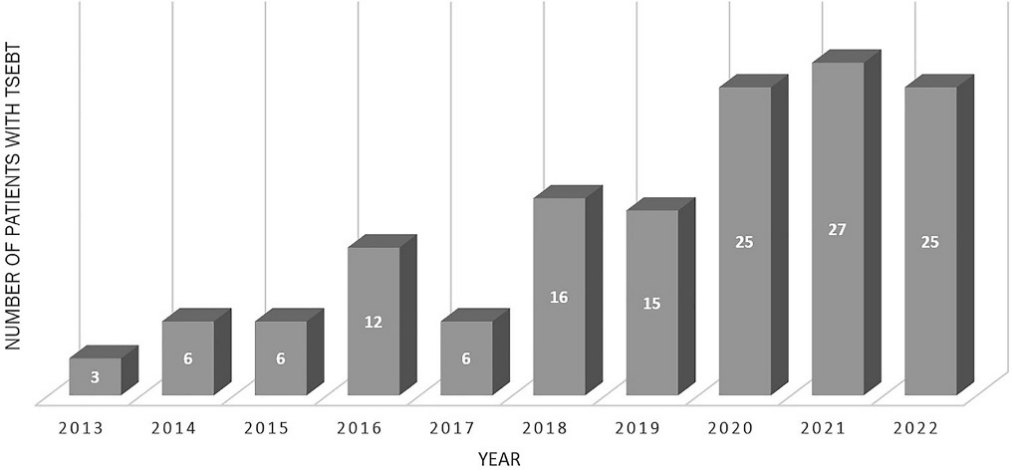
Number of patients undergoing total skin electron beam therapy (*TSEBT*) at Münster University Hospital

**Fig. 2:**
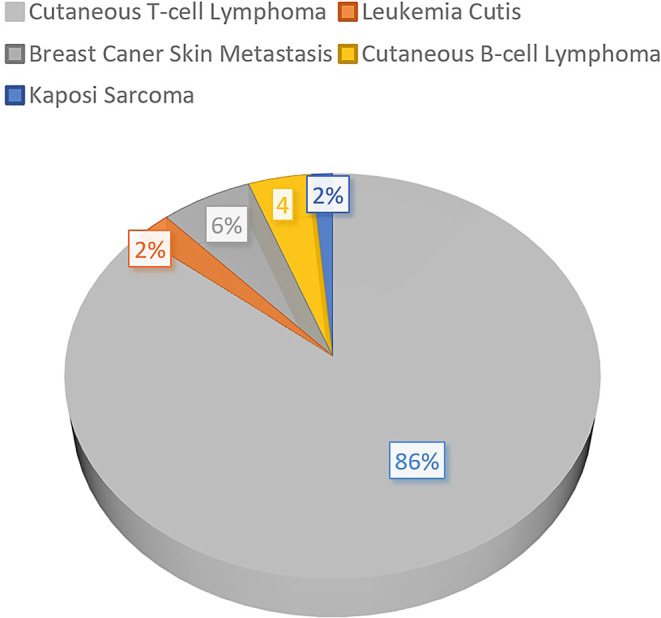
Indications for total (or partial) skin electron beam therapy at Münster University Hospital between 1988 and 2022 (*N* = 214)

The “six-dual-field” or modified Stanford technique is the most commonly used technique to deliver total skin electron beam therapy (TSEBT; [1, 2]). On the other hand, several institutions have also successfully used the rotational TSEBT technique [3–6]. The radiation treatment typically takes 20 min per fraction. Radiation dose distribution on the skin surface is usually assessed by thermoluminescent dosimeter (TLD) measurements [7, 8]. Supplementary local radiation to the underdosed areas, tumorous skin lesions, or pathologically enlarged lymph nodes may be applied to compensate for underdosing if clinically necessary. Based on the encouraging national and international data on reduced-dose TSEBT, dermatologists have had an increasing interest during the past decade in referring patients to radiotherapy (Fig. 1). Our technique has been previously described [7]. Indications for TSEBT usually include primary cutaneous T‑cell lymphoma (CTCL), Kaposi sarcoma, leukemia cutis, skin metastasis, and primary cutaneous B‑cell lymphoma with generalized skin involvement (Fig. 2).

## Role of TSEBT in the management of cutaneous T-cell lymphoma

The treatment algorithm for cutaneous T‑cell lymphoma (CTCL) is very complex and requires interdisciplinary decision-making [9, 10]. Radiotherapy (RT) is one of the most efficacious therapies for patients with primary CTCL due to its radiosensitivity [9]. Scholtz first employed RT to treat MF in the early 1900s [11]. However, the finding that most patients experience relapse outside of the radiation field argues in favor of systemic therapy to prevent relapse. Therefore since 1951, TSEBT has been used to treat diffuse CTLC involving more than 10% of the body surface area, although it remains a very specialized treatment that is not widely available [12]. The conventional 30–36-Gy regimen is time-consuming and is associated with significant treatment-related skin toxicities and late relapses [13–15]. Thus reduced-dose TSEBT regimens have been gaining interest recently with the hope of minimizing the risk of adverse events and the possibility for repetition in the event of relapse [14, 16–18]. In order to shorten hospital visits, a technique of once-weekly TSEBT (with a 4-Gy fraction) was employed at Memorial Hospital to a total dose of 32 Gy [19, 20]. However, low-dose RT (≤ 12 Gy) has yielded favorable results with a comparable overall response rate [14, 21–23]. In a toxicity analysis, the rate of RT-related adverse events was lower following low-dose regimens [24].

**Fig. 3:**
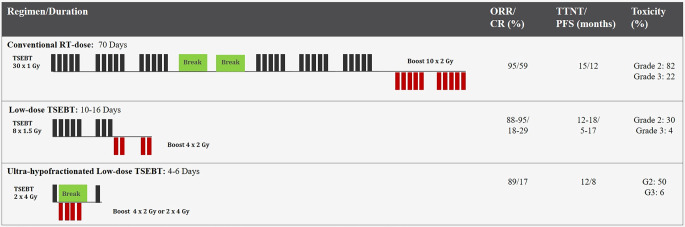
Course of total skin electron beam therapy (TSEBT) dose/fractionation and the clinical outcome of each regimen in cutaneous T‑cell lymphoma. *ORR* overall response rate, *CR* complete response, *TTNT* time to next treatment, *PFS* progression-free survival, *RT* radiotherapy

Following a total dose of 12 Gy, clinical response is achieved in almost all patients [18, 25]. However, the response duration is usually short [8, 17, 18]. In combination with maintenance therapy, TSEBT has been associated with improved outcomes [26, 27]. In a prospective German trial, ultra-hypofractionated TSEBT with 8 Gy in two fractions achieved reasonable disease control and symptom palliation with acceptable toxicity, greater comfort, and fewer hospital visits (Fig. 3; [28]). Concurrently and following TSEBT, immunotherapy has shown efficacy with a favorable safety profile [29–31]. Moreover, TSEBT improves patients’ symptoms and health-related quality of life within 2–4 weeks [27, 32, 33]. Furthermore, recent research indicates that TSEBT may improve peripheral blood involvement in patients with Sezary syndrome (SS) [34, 35]. Refractory skin manifestations from primary nodal non-Hodgkin lymphomas can also be treated with RT. However, careful consideration should be given to the skin toxicities associated with concurrent systemic therapies to TSEBT.

## Role of radiotherapy in the management of cutaneous B-cell lymphoma

In a long-term analysis of patients with cutaneous B‑cell lymphoma (CBCL; [36]), the 5‑year local control rate following low-dose focal RT is similar to conventional doses (86% vs. 90%, *p* = 0.4). Based on our experience, reduced-dose RT (4 Gy) might be applied in primary indolent CBCL, with the possibility of its repetition if required or dose-escalation (up to 24–30 Gy) in the case of refractory disease. Therefore, patients with CBCL and diffuse skin manifestations might require TSEBT instead of multiple local RT fields. Patients with primary indolent CBCL treated with TSEBT at Münster University Hospital with ≥ 12 Gy demonstrated an overall response rate of 100% [7].

## Role of radiotherapy in the management of leukemia cutis

The skin represents one of the sanctuary sites for residual leukemic cells after aggressive therapies. Leukemia cutis is a rare clinical leukemia presentation associated with a poor prognosis [37–40]. Therefore, patients are usually referred to RT after exhibiting progressive disease following different systemic treatment or stem cell transplantation. Reduced-dose TSEBT with 26 Gy is an effective treatment for controlling leukemia cutis progression. Lower TSEBT doses (12 Gy) might also be applied in palliative cases or in the case of concurrent systemic therapy.

## Role of radiotherapy in the management of Kaposi sarcoma

Owing to Kaposi sarcoma radiosensitivity, local RT is very effective for this type of disease [41]. In a randomized prospective trial, the conventional local RT dose of 24 Gy in 12 fractions was found to be safe as a hypofractionated regimen with 20 Gy in five fractions [42]. In patients with diffuse or generalized skin involvement, TSEBT can be applied to avoid the chasing technique [19, 43]. Furthermore, TSEBT in 4‑Gy fractions weekly to a cumulative dose of up to 32 Gy was very effective compared to the chasing technique using multiple local RT fields [19]. The efficacy of lower radiation doses in Kaposi sarcoma (< 20 Gy) remains questionable and warrants further investigation.

## Role of radiotherapy in the management of cutaneous metastases

In a meta-analysis of cutaneous metastases, the most common cutaneous metastases originate from advanced breast cancer and melanoma. Palliative local RT (normal fractionated or hypofractionated) for skin metastases is an effective and safe treatment with symptom reduction (i.e., fetor, secretions, or bleeding) and improvement in health-related quality of life [44]. Therefore, TSEBT, or partial skin electron beam therapy, can be indicated for patients with widespread skin metastases. The analgesic effect of RT is often observed at lower doses and achieves its maximum within a few weeks. In concomitant visceral metastases, systemic therapies are usually necessary and should be administered sequentially to avoid additional skin toxicity [45]. Consensus-based guidelines on integration of RT into targeted treatments for breast cancer are warranted.

## Conclusion

To sum up, TSEBT for skin manifestations of lymphoma, leukemia, Kaposi sarcoma, and metastases from solid tumors is a very effective treatment modality achieving a rapid reduction of disease burden and symptoms and improving health-related quality of life.
